# Assessment of Heart Rhythm and Arrhythmia Detection from Electromyographic Signals Using a Convolutional Neural Network

**DOI:** 10.17691/stm2025.17.6.01

**Published:** 2025-12-29

**Authors:** M.A. Sazhina, S.A. Lobov, A.S. Pimashkin, I.V. Loskot, V.B. Kazantsev

**Affiliations:** Research Engineer, Engineering Center; National Research Lobachevsky State University of Nizhny Novgorod, 23 Prospekt Gagarina, Nizhny Novgorod, 603022, Russia; DSc, Professor, Neurotechnology Department; National Research Lobachevsky State University of Nizhny Novgorod, 23 Prospekt Gagarina, Nizhny Novgorod, 603022, Russia; Leading Research Fellow, Laboratory of Neurobiomorphic Technologies; Moscow Institute of Physics and Technology (National Research University), 9 Institutskiy Pereulok, Dolgoprudny, Moscow Region, 141701, Russia; PhD, Senior Research Fellow, Research Laboratory of Stochastic Multistable Systems; National Research Lobachevsky State University of Nizhny Novgorod, 23 Prospekt Gagarina, Nizhny Novgorod, 603022, Russia; Assistant, Psychophysiology Department, Faculty of Social Sciences; National Research Lobachevsky State University of Nizhny Novgorod, 23 Prospekt Gagarina, Nizhny Novgorod, 603022, Russia; DSc, Head of Neurotechnology Department; National Research Lobachevsky State University of Nizhny Novgorod, 23 Prospekt Gagarina, Nizhny Novgorod, 603022, Russia; Head of the Laboratory of Neurobiomorphic Technologies; Moscow Institute of Physics and Technology (National Research University), 9 Institutskiy Pereulok, Dolgoprudny, Moscow Region, 141701, Russia

**Keywords:** electromyography, electrocardiography, heart rate variability, convolutional neural network, autoencoder

## Abstract

**The aim of the study** is to develop and validate an algorithm based on a convolutional autoencoder for assessing heart rhythm from surface electromyography (EMG) signals recorded by the EMG system Myosuit. The use of this system is intended to expand the capabilities for subsequent development of integrated systems for non-invasive monitoring of functional status (fatigue, stress, and cardiac arrhythmias).

**Materials and Methods:**

The study involved 6 healthy male subjects (mean age 21±2 years). Synchronous recording of EMG signals was performed using the EMG system Myosuit, the VNS-Micro electrocardiograph, and the Polar H10 monitor at rest, as well as during static and dynamic bicep contractions.

**Results:**

A fully convolutional autoencoder, trained on binary R-wave masks, was developed to extract the corresponding components of the cardiac cycles from the EMG recordings. The model’s performance was evaluated using the *F*-score in three scenarios: (i) on pooled data from all subjects; (ii) with one subject excluded from training and tested on their data (leave-one-subject-out); (iii) on the personal data of each subject individually. Based on EMG signals recorded from the pectoralis major muscle by the EMG system Myosuit sensors, the convolutional autoencoder demonstrated the feasibility of constructing a rhythmogram — a sequence of R–R intervals essential for heart rate variability analysis and arrhythmia screening. In Scenario (i), the maximum *F*-score was achieved for EMG signals at rest and during static bicep exercise; classification performance remained high during dynamic bicep exercise. The results of Scenario (ii) indicate that the system reliably operates when tested on data from a subject excluded from the training set. Scenario (iii) yielded the worst results. Given the algorithm’s high generalization capability for data not included in the training set, Scenario (ii) represents the most realistic application use case and demonstrates the best overall performance.

**Conclusion:**

The feasibility of using the universal EMG electrodes of the EMG system Myosuit for simultaneous monitoring of muscle activity and heart rate has been confirmed. The developed algorithm demonstrates high performance in the task of extracting a rhythmogram from EMG signals both at rest and during muscle load. This opens prospects for creating cost-effective wearable systems for the comprehensive assessment of functional status and real-time screening for cardiac arrhythmias without the need for a separate ECG channel.

## Introduction

The modern lifestyle, characterized by increasing physical inactivity and chronic stress, is a significant risk factor for a wide range of pathologies, including muscle hypertonia, psychosomatic disorders, and cardiovascular diseases [1]. The latter remains the leading cause of mortality, underscoring the critical importance of developing methods for early diagnosis and prevention. The analysis of heart rate variability (HRV) plays a key role in prenosological diagnosis and the assessment of an organism’s functional state [2]. The HRV method, based on measuring the time intervals between successive heartbeats (e.g., R–R intervals), is a highly sensitive tool for assessing the neurohumoral regulation of the heart and the balance between the sympathetic and parasympathetic nervous systems [3]. Changes in HRV parameters serve as a reliable indicator of general fatigue development and responses to stressors, finding application in both clinical practice and sports medicine [3, 4]. Furthermore, the analysis of R–R interval sequences (rhythmograms) forms the basis for the automated detection of cardiac arrhythmias [5].

Electrocardiography (ECG) is traditionally used to record the signal necessary for analyzing heart rate and its variability. However, the need for specialized electrodes and a separate recording channel can limit the use of ECG in settings involving long-term monitoring, athletic training, or daily activities, reducing user comfort and adherence to monitoring protocols.

Simultaneously, another widely used method — surface electromyography (EMG) — has also demonstrated potential for non-invasive cardiac monitoring. Its principle involves recording the bioelectric potentials of skeletal muscles. When electrodes are placed on the skin of the chest, the recorded signal represents a complex superposition of motor unit action potentials and a component arising from the heart’s electrical activity [6]. Historically, the cardiac component in EMG was considered an artifact that interfered with the analysis of muscle activity, and most research efforts were directed at its suppression using various methods: from frequency filtering [7] and adaptive filters [8] to more complex approaches such as independent component analysis [7, 9], wavelet transformation [6, 10], and singular spectrum analysis [11].

This study proposes to view the cardiac signal in EMG not as noise, but as a valuable diagnostic source of information. This opens the possibility of creating hybrid monitoring systems that use universal EMG electrodes to simultaneously assess neuromuscular and cardiac status. Such an approach eliminates the need for separate, specialized ECG equipment, which is particularly relevant for sports medicine, rehabilitation, and long-term preventive monitoring, where convenience and minimizing the number of sensors are important. Despite sporadic attempts to extract heart rate from mixed recordings (e.g., in [12], where EMG was used as an additional channel to reduce noise in classical ECG), this area of research remains underexplored.

Artificial neural networks (ANNs), particularly convolutional neural networks (CNNs), which have proven successful in pattern recognition tasks for biomedical signals [13], are the optimal tool for extracting rhythmograms from complex EMG signals. This study utilizes a convolutional autoencoder architecture — a network capable of learning a compressed representation of key features in the input data. U-Net-like architectures are often used to improve time-series segmentation [14], and the high-level Keras API is effectively used for rapid prototyping and deployment of such models [15].

**The aim of this research** is to develop and validate an algorithm based on a convolutional autoencoder for assessing heart rate from surface EMG signals recorded by the EMG system Myosuit thereby expanding the potential for creating integrated systems for non-invasive monitoring of functional state (fatigue, stress, and cardiac arrhythmias).

## Materials and Methods

The study was conducted at Lobachevsky State University of Nizhny Novgorod (Russia). The study design adhered to the standards set by the latest revision of the Helsinki Declaration and was approved by the Bioethics Committee of Lobachevsky State University of Nizhny Novgorod (protocol No.70, dated March 1, 2023). All participants provided written informed consent.

The study involved 6 healthy male volunteers (mean age 21±2 years). Inclusion criteria: absence of neurological, cardiovascular, or musculoskeletal diseases. Exclusion criteria: presence of implanted electronic devices, skin diseases at electrode placement sites.

The following equipment was used to record bioelectric cardiac signals:

the EMG system Myosuit a garment-integrated system for monitoring muscle activity — an experimental development of the Department of Neurotechnology at Lobachevsky State University of Nizhny Novgorod [16–18];
the VNS-Micro electrocardiograph (Neurosoft, Russia) as the reference standard for ECG;
the Polar H10 heart rate monitor (Polar Electro Oy, Finland).

**Figure 1.**
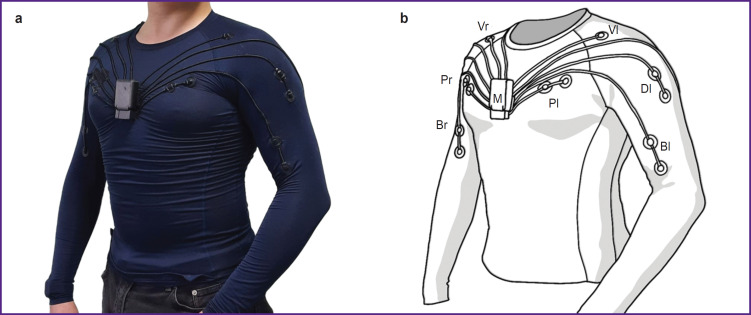
External view of the EMG system Myosuit: (a) overall appearance of the suit; (b) main garment elements with sensors, including channels for *musculus biceps brachii* (Br — right, Bl — left), *musculus pectoralis major* (Pr — right, Pl — left), *musculus deltoideus* (Dl — left); vibromotors (Vr — right, Vl — left); M — removable signal acquisition unit

The EMG system Myosuit is a wearable device for monitoring and visualizing human muscle activity based on compression gear with integrated electromyographic sensors (Figure 1). The system supports 8 channels for recording EMG signals, with electrodes positioned symmetrically on the right and left sides over the following muscles: *musculus biceps brachii*, *musculus triceps brachii*, *musculus deltoideus*, and *musculus pectoralis major*.

In preliminary data visualization experiments using the EMG system Myosuit rhythmic peaks were observed in the EMG from the left *musculus pectoralis major*, presumably originating from the heart’s electrical activity.

In the first stage of the study, to determine the feasibility of using the EMG system Myosuit as a recorder of bioelectric potentials from both skeletal musculature and cardiac activity, simultaneous data recording was performed using the EMG system Myosuit sensors, the VNS-Micro electrocardiograph, and the Polar H10 chest monitor. Data were recorded using proprietary software ech_monitor_v1_5, as well as Poly-Spectrum.NET (v. 6.0.9) and Polar Sensor Logger, respectively.

**Figure 2.**
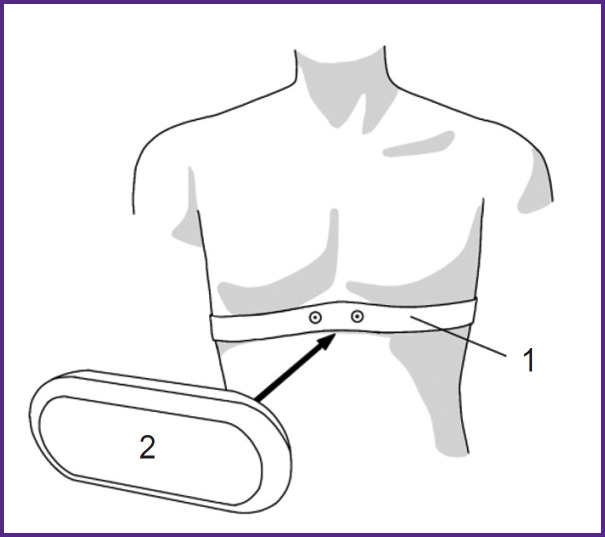
Schematic of Polar H10 sensor placement: *1* — strap with electrodes, *2* — signal acquisition unit

The Polar H10 monitor consists of a strap with electrodes and a sensor unit that transmits data via Bluetooth to a paired receiving device (Figure 2).

Prior to recording, device preparation was carried out: moistening the area of the Polar H10 monitor strap where the electrodes are located; applying the strap around the chest, ensuring a tight fit; and attaching the sensor unit. Next, the electrodes of the EMG system Myosuit were treated with alcohol, the suit was donned by the subject, gel was applied to the electrode contact surfaces, and the signal acquisition unit was connected. Subsequently, electrodes of the VNS-Micro electrocardiograph were placed on the subject’s upper limbs according to the standard and augmented limb lead scheme, with prior skin defatting using alcohol for better electrode contact and application of contact gel to the electrode sites.

The experimental protocol included recording data during the following conditions:

at rest (standing) for 10 s;
during a static biceps exercise, holding dumbbell bars (1.5 kg) with arms bent for 10 s;
during a dynamic biceps exercise with 6.5 kg dumbbells for 3 sets of 10 repetitions, with a 60-second rest interval between sets;
during a static biceps exercise, holding dumbbell bars (1.5 kg) with arms bent for 10 s;
at rest (standing) for 10 s.

### Statistical data processing

Data processing and analysis were performed using Python 3.11.9 and the Keras 3.9.2 library. The quality of R-wave recognition by the autoencoder was assessed by calculating the *F-*measure [19] based on precision (*P*) and recall (*R*) values obtained from the classification results:

P=TPTP+FP,R=TPTP + FN,

where the number of neural network activations was counted separately for true positives (TP), true negatives (TN), false positives (FP), and false negatives (FN). The *F-*measure was defined as:

F=2PRP+R.

The analysis was conducted under three data processing scenarios:

Scenario (i): training on 80% of the combined data from all subjects, testing on the remaining 20%.
Scenario (ii): training on data from all subjects except one, testing on the data of the excluded participant. This procedure was repeated for each subject (leave-one-subject-out cross-validation).
Scenario (iii): training and testing only on individual data from each subject. Each subject’s data were split into 80% training set and 20% test set. Training and testing did not include data from other subjects. The procedure was repeated for each participant.

## Results

**Figure 3.**
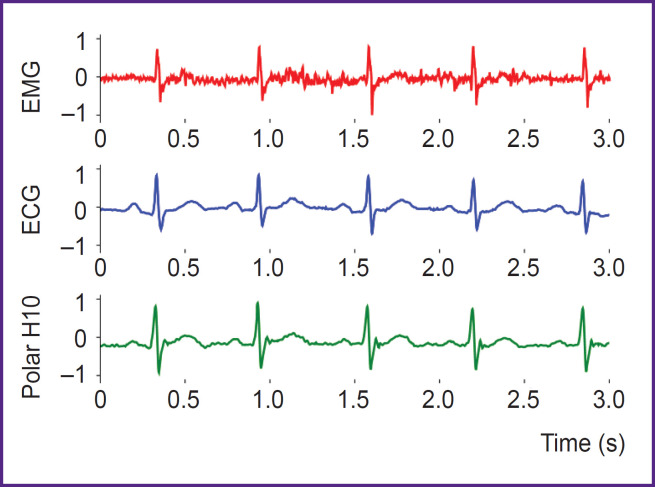
Aligned signals recorded using the EMG system Myosuit, Polar H10 monitor, and VNS-Micro electrocardiograph (subject was at rest) EMG — electromyography, ECG — electrocardiography

Synchronous recording of signals using the EMG system Myosuit the VNS-Micro electrocardiograph, and the Polar H10 monitor confirmed the presence of a cardiac component in the EMG signal recorded from the left *musculus pectoralis major*. As shown in Figure 3, which displays normalized signals from all three systems, the peaks of the heart’s bioelectrical activity coincide in the recordings, providing visual confirmation of the possibility of detecting R-waves directly through EMG electrodes. This observation is consistent with previous studies [20] that considered the ECG component as noise relative to the EMG signal. Specifically, it has been noted that when using electrodes of sufficient area in this anatomical region, the recorded signal represents a superposition of EMG and ECG components [20]. The obtained data indicate the fundamental possibility of recording R-waves and subsequently constructing a rhythmogram using universal EMG electrodes without the need for a dedicated separate channel.

To automate the detection of ECG R-waves in EMG signals, a convolutional autoencoder was developed using the Keras API. An autoencoder is a neural network with two components: an encoder block, which transforms input data into a lower-dimensional latent space, and a decoder block, which reconstructs the original data from this compressed form. By training to minimize reconstruction error, the autoencoder prioritizes significant features (in this case, R-wave patterns) and filters out noise.

**Figure 4.**
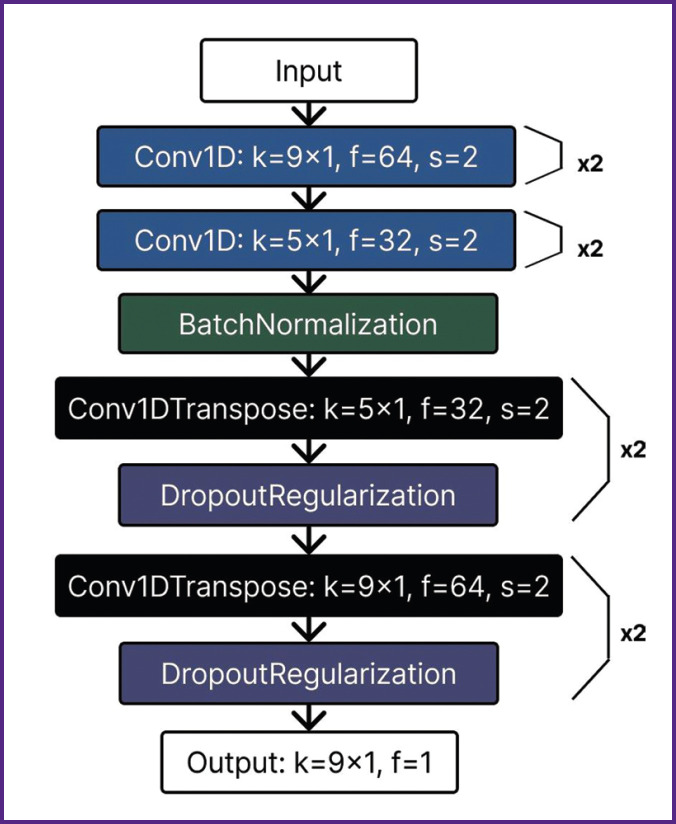
Architecture of the developed autoencoder: Conv1D — one-dimensional convolutional layer; Conv1DTranspose — one-dimensional transposed convolutional layer; k — kernel size; f — number of filters; s — stride size; BatchNormalization — batch normalization; DropoutRegularization — dropout layer

The core architecture of the developed autoencoder (Figure 4) consists of one-dimensional convolutional layers (Conv1D), specifically adapted for processing EMG signals. Unlike traditional two-dimensional convolutions used in computer vision, a one-dimensional layer analyzes time series data, enabling efficient detection of key signal shape features (peaks of various polarities) critical for R-wave identification.

To balance global context and local precision, the architecture combines large kernels (size 9×1) to capture a wide signal window and small kernels (size 5×1) for precise R-wave localization. The model optimization process involved fine-tuning key hyperparameters such as the number of convolutional layers, convolution kernel sizes, and the number of filters in each layer. Bayesian optimization methods were applied to achieve an optimal balance between maximum R-wave extraction accuracy and model latent space complexity. Dimensionality reduction of feature maps in the encoder block was implemented using strided convolutions (stride=2) to reduce computational costs compared to traditional pooling layers. Batch normalization was applied after convolutional layers in the encoder block to stabilize the training process and accelerate convergence.

The decoder block utilized transposed convolutional layers (Conv1DTranspose) to reconstruct the signal from the compressed representation while minimizing interpolation artifacts. A dropout layer was integrated into the architecture to prevent overfitting.

**Figure 5.**
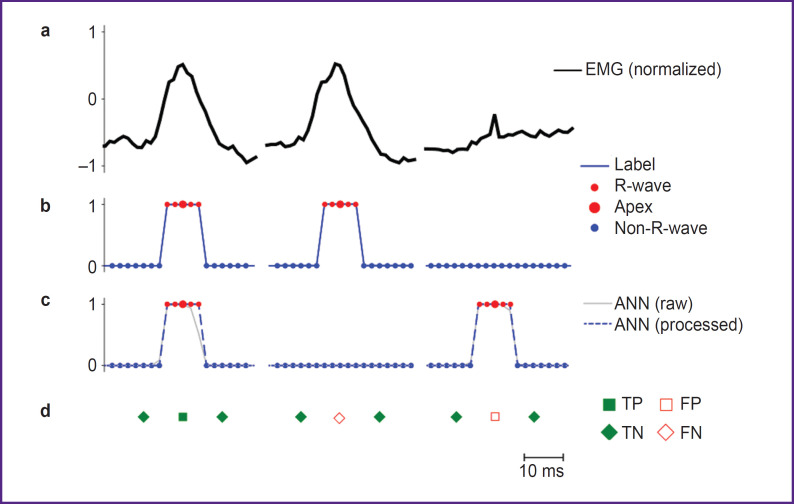
Example of electromyography (EMG) data labeling and assessment of artificial neural network (ANN) response correctness: (a) normalized electromyogram; (b) manual labeling of R-waves across five time samples, with the central point corresponding to the R-wave apex; (c) examples of the ANN output signals; (d) detection of true positive (TP), true negative (TN), false positive (FP), and false negative (FN) responses of the ANN

Although autoencoders are typically trained in an unsupervised manner, this study implemented supervised learning on labeled data using the backpropagation algorithm. The data labeling strategy (Figure 5) included features specific for R-wave detection. EMG was recorded while subjects performed physical exercises or during rest periods, with each recording corresponding to one set (or rest interval) lasting from 13 s to 1.5 min at a sampling rate of 500 Hz. After recording, data were normalized to the range [–1, 1] (Figure 5 (a)) and fragmented into samples of 1600 points for network input.

The label vector was implemented as a binary mask (Figure 5 (b)), where each R-wave was marked by five consecutive ones centered at its apex. All other points were marked as zeros. The network output corresponds to the input dimension (1600 points), with values in the range [0, 1] and classified as belonging (≥0.5) or not belonging (<0.5) to an R-wave (Figure 5 (c)).

For subsequent classification quality assessment, each sequence of five ones in the label vectors and network outputs was interpreted as a single event — the presence of an R-peak. A true positive (TP, Figure 5 (d)) was defined as a match between a predicted sequence and a sequence in the labels. Predicted peaks without corresponding labels were classified as false positives (FP), and missed labels as false negatives (FN). True negative (TN) responses were calculated as the number of zero points matching in the labels and model output, divided by the event duration (5 points) for time normalization.

**Figure 6.**
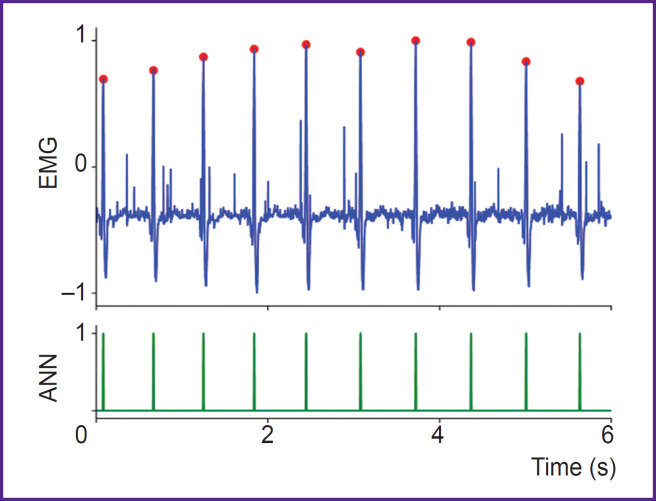
Example of R-wave detection in the EMG signal from the left *musculus pectoralis major* at rest ANN — output signal of the artificial neural network, EMG — electromyography

Figure 6 shows an example of an EMG signal from the left *musculus pectoralis major* recorded at rest (subject standing upright) and the results of the autoencoder classification. In this position, the pectoral muscles are relaxed, and the background EMG signal has low amplitude, facilitating reliable R-wave detection by the neural network. As a result, the ANN achieves near-perfect recognition accuracy (100% in most cases). This indicates that data relevant for HRV can indeed be extracted from the EMG signal of the left pectoral muscle at rest.

**Figure 7.**
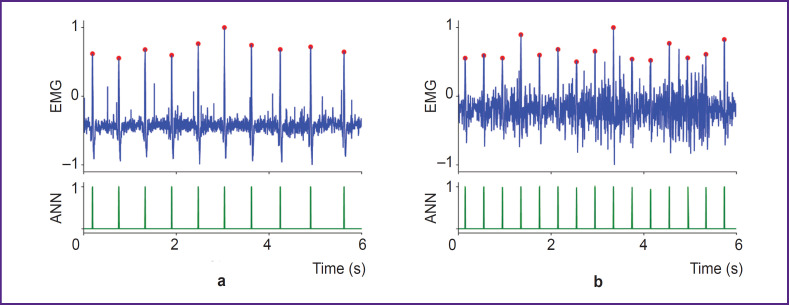
Example of R-wave detection in the EMG signal from the left *musculus pectoralis major* during a biceps exercise: (a) isometric tension; (b) dynamic exercise. ANN — output signal of the artificial neural network, EMG — electromyography

However, this finding does not hold for all real-world training scenarios involving physical activity. We found that the proposed method cannot be applied during active work of the pectoral muscles, such as during push-ups or bench presses. In these exercises, the EMG signal amplitude increases sharply, overwhelming the R-waves. On the other hand, such high-intensity pectoral exercises usually constitute only a part of a training program. In most training situations, other muscle groups (e.g., limbs or core) dominate, while the pectoral muscles play a secondary stabilizing role. Applying the proposed approach to EMG recordings from the left pectoral muscle during static (Figure 7 (a)) and dynamic (Figure 7 (b)) exercises targeting other muscles (e.g., biceps) shows that reliable R-wave extraction is possible in such cases.

Statistical analysis of classification quality based on the *F-*measure, conducted across different data processing scenarios for the 6 subjects, confirms the possibility of reliable R-wave (and, consequently, R–R interval) recognition by the autoencoder (see the Table).

**Table:** *F-*measure values for predicting R-wave positions in different data processing scenarios

Scenario	Train samples	Test samples	Subjects’ training context			
			Rest	Static exercise	Dynamic exercise	All contexts
I	80% of all subjects (St)	20% of St	**1.00**	**1.00**	**0.94**	**0.97**
II	St–S1	S1	0.96	1.00	1.00	1.00
	St–S2	S2	1.00	1.00	0.95	0.97
	St–S3	S3	0.82	0.99	0.99	1.00
	St–S4	S4	1.00	1.00	0.89	0.94
	St–S5	S5	1.00	0.93	0.97	0.94
	St–S6	S6	1.00	0.98	0.89	0.94
	Median		**1.00**	**1.00**	**0.95**	**0.97**
	Mean		0.97	0.99	0.95	0.97
III	80% of S1	20% of S1	0.89	0.83	0.87	0.85
	80% of S2	20% of S2	0.96	1.00	0.75	0.89
	80% of S3	20% of S3	0.96	0.91	1.00	1.00
	80% of S4	20% of S4	0.82	0.93	0.87	0.98
	80% of S5	20% of S5	0.89	0.75	0.92	0.96
	80% of S6	20% of S6	1.00	0.85	0.89	0.95
	Median		**0.92**	**0.88**	**0.88**	**0.96**
	Mean		0.92	0.88	0.88	0.94

N o t e: St — all subjects, S — each subject.

Scenario (i), using pooled data from all subjects (80% training, 20% test), allows for an assessment of the overall ANN performance. Notably, classification achieved maximum quality (*F*=1) for signals recorded at rest and during the static biceps exercise and demonstrated sufficiently high accuracy for the dynamic biceps exercise (*F*=0.94).

Scenario (ii) models the use of the ANN in the EMG system Myosuit under real-world conditions, requiring the model to be trained on a large database of existing users to classify data from new users not included in the training set. Accordingly, in this scenario, training was performed on data from all subjects except one, and testing was performed on the excluded participant, repeating this procedure for each subject. The classification results (median *F-*measure values: at rest *F*=1, static exercise *F*=1, dynamic exercise *F*=0.95) were no worse than those using pooled data from all subjects, indicating the readiness of the proposed approach for real-life application.

Training the ANN on personal datasets (Scenario (iii), 80% training, 20% test) unexpectedly yielded the worst results (mean values: *F*=0.92 at rest, *F*=0.88 for static, and *F*=0.88 for dynamic exercises). This degradation in classification quality is likely associated with the limited size of individual training sets, leading to overfitting of the ANN.

Thus, considering the algorithm’s generalization capability for data from new, unseen subjects, Scenario (ii) represents the most realistic use case and demonstrates the best overall results.

## Discussion

The obtained results demonstrate the fundamental feasibility of solving a relevant medical task — non-invasive heart rate monitoring — using a non-traditional signal source, namely surface EMG recorded by the multi-channel EMG system Myosuit. A key innovation of this work is not the suppression of the ECG component as an artifact in data assessment, but its targeted extraction for subsequent rhythmogram analysis and HRV parameter calculation. This approach allows universal EMG electrodes located on the pectoralis major muscle to be considered as multifunctional sensors capable of simultaneously recording muscle activity and heart rate. Eliminating the need for additional specialized cardiomonitoring equipment opens significant opportunities for the development of wearable technologies in sports and preventive medicine.

An important achievement of the work is the development and successful testing of a specialized convolutional autoencoder architecture adapted for processing one-dimensional EMG time series. The use of one-dimensional convolutional layers with kernels of different sizes allows for effective capture of both the global signal context and the local features of R-wave shape. The high *F-*measure values achieved in various scenarios indicate that the autoencoder learned to reliably identify the cardiac component even against a background of muscle activity.

Of particular interest for practical implementation is the analysis of the ANN’s generalization capability. The best results, shown in Scenario (ii) (training on a group of subjects with testing on a new participant), indicate that the proposed autoencoder can extract universal R-wave features that are invariant to individual signal characteristics. This is a significant advantage for clinical and applied use, as it allows a pre-trained model to be applied to new users without the labor-intensive process of collecting and labeling personal data. The lower effectiveness achieved in Scenario (iii) (training on data from a single subject) highlights the risk of overfitting when working with small data volumes and confirms the advisability of using group models for widespread implementation.

Analysis of existing methods for cleaning EMG from ECG artifacts [6–11] shows that these approaches primarily solve the inverse problem — suppressing the cardiac component to obtain a “clean” myographic signal. Our proposed machine learning method is focused on the inverse task of extracting the rhythmogram, opening new possibilities for integral physiological assessment. The operation mechanism of the convolutional autoencoder can be interpreted as a nonlinear adaptive filter that does not merely attenuate the signal in a certain frequency band but actively recognizes specific R-wave patterns based on their shape and temporal characteristics, representing a more robust approach under conditions of varying muscle activity levels.

The obtained results could play a key role in developing monitoring systems for specific medical and paramedical fields.

1. Sports medicine. The joint analysis of EMG parameters (muscle fatigue) and HRV (strain on regulatory systems) in real-time could form the basis for creating intelligent systems that provide early warning of overtraining and adverse functional states in athletes.

2. Preventive medicine and rehabilitation. The technology allows for long-term, user-unobtrusive monitoring of stress and general functional state via HRV during daily activities, as well as during rehabilitation after illnesses.

3. Screening for rhythm disorders using “smart clothing” elements. The derived rhythmogram is suitable for detecting episodes of tachycardia, bradycardia, and potentially other types of arrhythmias in situations where the use of a standard ECG monitor is inconvenient or impossible.

One limitation of the method is the dependence on the level of muscle tension. As the results showed, during periods of high pectoral muscle activity (e.g., during push-ups), the EMG signal amplitude significantly exceeds that of the ECG component, complicating its extraction. However, given that in many training and daily life scenarios the pectoral muscles play a stabilizing rather than a primary role, the proposed algorithm can be applied to a wide range of loads on other muscle groups, as confirmed by the successful detection of R-waves during biceps exercises.

For the further development of technology for creating integrated systems for non-invasive functional state monitoring, promising directions include: expanding the dataset by including a larger number of subjects with varying fitness levels and individuals with diagnosed rhythm disorders to validate the method in clinical settings; adapting the model to work with signals recorded from other muscle groups; integrating the algorithm into the EMG system Myosuit software for online signal processing and real-time HRV calculation.

An additional area of interest is investigating the algorithm’s robustness to various types of artifacts, such as electrode motion or respiratory activity.

Overall, this work lays the foundation for creating a new class of hybrid physiological monitoring systems that combine the assessment of neuromuscular and cardiac components of a human’s functional state, which is highly relevant in the context of modern personalized medicine development.

## Conclusion

The fundamental feasibility of recording and automatically extracting a rhythmogram from surface EMG signals from electrodes placed on *musculus pectoralis major* has been experimentally confirmed, as demonstrated using the EMG system Myosuit. The developed convolutional autoencoder demonstrated high efficiency in automatically extracting R-waves from the mixed EMG signal under resting conditions and during moderate physical exertion, as confirmed by high *F-*measure values.

The hypothesis that universal EMG electrodes can be used for simultaneous monitoring of muscle activity and key cardiological parameters — heart rate and its variability — has been confirmed. This opens direct prospects for creating integrated wearable systems that provide a comprehensive assessment of functional state (fatigue, stress, and basic rhythm disorders) in real-time without the need for additional specialized ECG sensors.

The obtained results are significant for use in applied fields such as sports medicine, where monitoring an athlete’s state “here and now” is crucial, and preventive medicine, which focuses on long-term health monitoring.

Further development of this work involves adapting the algorithm for online processing, its validation on broader and more diverse subject groups, including patients with cardiac pathology, and integration into the practice of non-pharmacological functional state control.
